# An Exploration of Family Caregiver Experiences of Burden and Coping While Caring for People with Mental Disorders in Saudi Arabia—A Qualitative Study

**DOI:** 10.3390/ijerph17176405

**Published:** 2020-09-02

**Authors:** Loujain Sharif, Shimaa Basri, Fidaa Alsahafi, Mashael Altaylouni, Shihanah Albugumi, Maram Banakhar, Alaa Mahsoon, Nofaa Alasmee, Rebecca J. Wright

**Affiliations:** 1 Department of Psychiatric and Mental Health Nursing, Faculty of Nursing, King Abdulaziz University, Jeddah 21551, Saudi Arabia; nalasmee@kau.edu.sa; 2 Faculty of Nursing, King Abdulaziz University, Jeddah 21589, Saudi Arabia; Shimaa.basri98@gmail.com (S.B.); Fidaaalsahafi1998@gmail.com (F.A.); Mashaelrt@hotmail.com (M.A.); Shehanh1998.911@gmail.com (S.A.); 3 Department of Public Health Nursing, Faculty of Nursing, King Abdulaziz University, Jeddah 21551, Saudi Arabia; ahbbanakher3@kau.edu.sa (M.B.); mahsoon@kau.edu.sa (A.M.); 4 Johns Hopkins School of Nursing, Baltimore, MD 21205, USA; Rebecca.wright@jhu.edu

**Keywords:** caregivers, caregiver burden, coping, mental disorder

## Abstract

Family caregivers of people with mental disorders face a number of burdens and stressors, such as associative stigma and burnout. These burdens are often a result of their caring role coupled with insufficient support or ineffective coping strategies, which can affect their quality of life and biopsychosocial integrity that, in turn, may affect the care they provide. This study aimed to explore the experiences of family caregivers of people with mental disorders, through examining the burdens that they face and the coping strategies that they use. Using a descriptive qualitative approach, 13 semi-structured interviews were conducted with members of the Saudi public, recruited through popular social media platforms and analyzed using thematic analysis. Five main themes were constructed from the data: Type of care, Challenges, Coping and support, Perceptions of public awareness, and Messages to others. The findings emphasize the different types of burdens that caregivers experience, and their needs that require a range of responses such as educational training on effective coping strategies, and psychological support in the form of counseling or group therapy. This study highlights the voice of caregivers and their message to the public, in order to correct the misconceptions surrounding mental disorders and those associated with them.

## 1. Introduction

Globally, the burden of mental disorders is on the rise, impacting socio-economic status, health, and human rights [1]. In this paper we draw from the DSM-5 definition of mental disorders: “a mental disorder is a syndrome characterized by clinically significant disturbance in an individual’s cognition, emotion regulation, or behavior that reflects a dysfunction in the psychological, biological, or developmental processes underlying mental functioning” which includes neurocognitive disorders such as Alzheimer’s and autism [2]. The burden of mental disorders affects not only those living with them, but also those who care for them, from healthcare professionals to family caregivers. A primary caregiver is defined as: “A member of the family who is most involved with the care of the outpatient” [3]. Universally, deinstitutionalization has led to a shift in care roles from healthcare professionals—particularly nurses—providing primary care in institutions to a more integrated, community-based approach with family members now acting as primary caregivers [4]. Since the deinstitutionalization movement, it has been estimated that the provision of care for 50–90% of people with mental disorders has now shifted to the responsibility of family members [5]. Research has also reported that the degree of responsibility carried out by family caregivers varies, depending on the severity of the disorder of the person with the mental illness [6]. However, typical responsibilities include providing emotional support such as active listening, physical company, and assisting with daily living activities such as grooming and personal hygiene [7]. Although deinstitutionalization has mostly brought about positive outcomes, it is not without shortcomings wherein family caregivers may be unprepared to manage unpredictable behaviors, such as violence and verbal or physical aggression, which have all been found to be contributing factors toward caregiver burden [8]. Family caregivers experience distinct types of burdens that can disrupt their biopsychosocial integrity, including physical, psychological, social, and economic burdens. Physical burden can be caused by the significant side effects of certain psychotropic drugs (e.g., antipsychotics) [9,10] that restrict or impact the patient’s ability to mobilize and provide self-care, requiring additional physical support from the caregiver. This can lead to an over-dependency on caregivers by people with mental disorders leading to exhaustion and burnout [11,12,13,14]. Psychological burden is usually expressed as grief and depression [11,12,13,14], for example, in a study focusing on female spousal caregivers, it was found that female spouses caring for husbands with severe mental illness typically experience emotional detachment and psychological distress. The results of this study stressed the different types of emotional burdens, entailing emotional detachment, emotional distancing, mutual hostility, pity instead of love, emotional exhaustion, despair and hopelessness, feelings of incompetence and exhaustion, loss of identity, interest and motivation, being trapped in the various roles, and being a scapegoat [13]. Social burden, which can be demonstrated as adverse effects on lifestyle, restriction in routine, harnessing social relationships and support, dysfunctional family process, and conflict between family members, can lead to dissatisfaction and fear of social isolation [11,12,13,14]. Furthermore, economic burden results from caregivers having to deal with role shifts, such as being the primary caregiver and breadwinner [11,12,13,14], in addition to financial strains for example, costs of psychotropic medications and limited coverage of free treatment as stipulated by governmental policies [10]. Family caregivers of people with mental disorders use a variety of coping strategies to manage the stress and burden of caregiving. These coping strategies have been linked to factors such as caregiver-expressed feelings, social support, and the value and quality of life of caregivers [7]. Although there have been many studies on the experiences of family caregivers of people with mental disorders, less attention has been paid to the significant distinctions between countries. Yet, it has been well-established that cultural differences and related factors can affect public perceptions and attitudes about those with mental disorders and, in turn, may cause different experiences in caregivers [15]. Globally, family caregivers face problems and stressors related to caring for their relatives with mental illnesses, such as stigma and ineffective coping strategies, where some of them are not ready to undertake their caregiving roles. This is indeed the case in Saudi Arabia [16]; according to the first Saudi National Health and Stress Survey, conducted on a sample of 4004 individuals in Saudi Arabia examined the occurrence of mental health conditions. The statistics demonstrated that 34% of Saudis met the criteria for a mental health condition, where these criteria were met by 36% of Saudi females and 33% of Saudi males; however, only 5% of the population had sought treatment [17]. Therefore, it is important to focus on family caregiver experiences, in order to explore their needs and preparedness to care for their loved ones who are suffering from mental disorders. Additionally, there may be cultural variations in caregiver burdens and challenges and interventions need to be tailored accordingly. For example, previous research identified unique perceptions of the cause and nature of mental disorders within Saudi culture, such as the commonly shared belief of the evil eye and its negative influence on mental wellbeing [18]. If the problems and stressors of caregivers are not overcome, they may inevitably lead to burnout and exhaustion, an increase in burden, a lack of social support, high levels of stigma, and a decrease in quality of life [19]. Most of the previous research conducted in this area used quantitative research methods to assess the psychological and socio-economic status of the caregiver. Quantitative research is potentially a limiting factor for their results, as it lacks rich detail. Furthermore, to date, there have only been very limited published research studies on this topic within the Saudi context. Hence, we conducted this study to provide more in-depth information about the experiences and challenges of caregivers, which can contribute to suggesting solutions to overcome these challenges. This study aimed to explore the experiences of family caregivers of people living with various mental disorders through examining the burdens that they face and the coping strategies that they use.

## 2. Materials and Methods

### 2.1. Study Design

This study adopted a descriptive qualitative research design, using semi-structured interviews with family caregiver participants caring for relatives with mental illnesses. This approach was deemed appropriate for the research aim as the primary goal of qualitative descriptive research is to provide a comprehensive inductive summarization of specific events experienced by a group of people, in terms of themes that are described in an elucidated manner [20]. 

### 2.2. Ethical Considerations

The research protocol was approved on 01/03/2020 by the Nursing Research Ethical Committee of a Public University (NREC Serial No: Ref No 1M. 24). The study was conducted in accordance with the Declaration of Helsinki. Prior to the interview, participants were given a full explanation about the research aim, the researcher’s responsibilities, the potential risks and benefits of participating, and their right to withdraw from the study at any time without any penalty. Participants were also made aware of the members of the team who would have access to their interview transcripts. Each participant was fully aware, and understood the essence of the study and agreed to take part. This study was conducted during the initial height of COVID-19 thereby preventing collection of written consent. At this stage, as many participants did not have capacity to provide digital signatures, the NREC gave permission to obtain verbal consent which was captured at the start of the audio recorded interview. Each participant caregiver was anonymized and identified by a code “Caregiver = (C),” followed by a unique identifier number. The research team was comprised of nine members; a senior, doctoral-trained nurse researchers: (M.B., A.M., and R.W.): two with expertise in mental health (L.S. and N.A.), and a team of student researchers (S.B., F.A., M.A., and S.A.) who received formal training in qualitative research methods including data collection and analysis. The senior research team oversaw the design and conceptualization of the research project while the student researchers supported data collection and initial analysis. 

### 2.3. Study Setting and Participant Recruitment

This study was conducted in Saudi Arabia. According to the most recent statistics listed by the Ministry of Communication and Information Technology, Facebook and Twitter are considered to be the most popularly used forms of social media at present in Saudi Arabia [21]. Therefore, a purposive sampling strategy was used across these social media platforms to recruit the target participants, which enabled the researchers to reach the family caregivers of people with mental disorders from different cities across Saudi Arabia. Specifically, an advertisement calling for participants and an initial screening survey was constructed, using Google Forms to recruit potential participants. Participants who responded to the advertisement, completed the screening survey, and met the inclusion criteria were then contacted by the researchers to arrange an interview date and time. Criteria for inclusion for study participants included self-reported living with a person with a mental illness, providing direct care to the individual, having been diagnosed by a psychiatric or mental healthcare provider, and being an Arabic or English speaker.

### 2.4. Data Collection Procedure

Data were collected by (S.B., F.A., M.A., and S.A.) using audio recorded, semi-structured interviews conducted via telephone or virtual communication platforms as per the participants preference in accordance with recent governmental regulations regarding social distancing, to maintain the safety of participants and the researchers during the Coronavirus disease of 2019 (COVID-19) pandemic. On average, the interviews lasted between 25 and 46 min. Semi-structured interviews were identified as the most appropriate data collection method for this design as they (1) enabled focused discussion with scope for open-ended questions, and (2) increase rigor in reliable and comparable qualitative data collection when interviews are conducted by multiple researchers [22]. The student researchers (S.B., F.A., M.A., and S.A.) used a semi-structured interview guide with open-ended questions and prompts to guide the participants. The interview guide was developed by the lead author (L.S) based on the challenges experienced by caregivers observed in the literature and clinical observations. The interviews examined the context of the experience of having to care for people with mental disorders by exploring the caregiver’s burdens, coping strategies, and experiences of stigma. Specifically, questions focused on the types of care that were provided and the challenges that were faced, combined with care, associative stigma, and issues related to disclosure, as well as the effect of the public opinion on their experiences. The student researchers also made field notes during the interviews, which were transcribed and used to provide additional context during data analysis. Of the 13 interviews conducted, 12 were in the participant’s native language (Arabic) and one interview was conducted in the English language, as per the participant’s preference. Participants also agreed to follow-up contact if the research team had additional questions and clarifications during the analysis process.

### 2.5. Data Analysis

Data were managed using Microsoft Word and Apple Pages. Data analysis was guided by Braun and Clarke’s six-phase process of thematic analysis (1. familiarize yourself with the data, 2. generating initial codes, 3. searching for themes, 4. reviewing themes, 5. defining and naming themes, 6. producing the report) [23]. In phase 1, all interview audio recordings were downloaded and then transcribed verbatim by the research team. The interviews were transcribed initially in Arabic, and then translated into English for data analysis by five members (S.B., F.A., M.A., S.A., and L.S) of the research team who are bilingual. To confirm that the interviews were correctly and accurately transcribed, the researchers reviewed all interviews twice. Participants were ascribed identifiable codes to maintain their anonymity and to allow for the management of their data. Using an inductive approach [24], the researchers individually familiarized themselves with the data by reading and re-reading the interview transcripts, searching for broad themes and gathering data across the transcripts. In phase 2, following an in-depth review of the first four interviews, under the guidance of the lead author, the research team gathered to discuss early data findings and initial codes. In phase 3, through a process of discussion and reflection of those interview data, a coding tree was developed and potential themes and sub-themes identified. In phase 4, the researchers then, independently, returned to the remaining nine interviews and using the coding tree continued the process of thematic analysis to identify themes and sub-themes. In phase 5, the research team met again when all interviews had been analyzed to further verify and discuss the identified themes and subthemes and agree upon the final definitions and names. Data saturation (the point at which no new themes are identified connected to the phenomena under investigation) [25] was initially noted at the fourth interview. Saturation was confirmed by the eighth interview and further verified across the remaining five interviews, with provision of additional examples that were representative of the themes and sub-themes. All discrepancies or disagreements in the process of data analysis and with regard to the themes and naming of the themes generated were discussed as a team under the supervision of the supervising researchers until resolution was reached. In addition, as per the protocol, three participants were contacted for clarification and additional context to ensure that resolution of discrepancies was in line with participants experiences and not biased by the perspectives of the research team. In preparation for phase 6, verbatim quotes of the participants were selected to illustrate the identified themes and sub-themes. 

## 3. Results

### 3.1. Participant Characteristics 

Fourteen people responded to the recruitment materials. After reviewing for eligibility, 13 Saudi caregivers were included and completed interviews. Participant characteristics are summarized in Table 1. Approximately half of the caregivers were single and the majority (77%) were female, with overall caregiver ages ranging between 21 and 65 years. Most of the primary caregivers were students, with only three in current employment. Caregivers varied in their relationship to the individual with the mental disorder, with 38% being a sibling, 31% a parent, 15% a child, 8% a spouse, and 8% other (i.e., uncle). The most common types of mental disorders among the individuals that were being cared for were depression and bipolar. A total of 77% of caregivers shared their caregiver role, and the remaining 23% served as primary caregivers, responsible for all aspects of the care provided.

**Figure 1:**
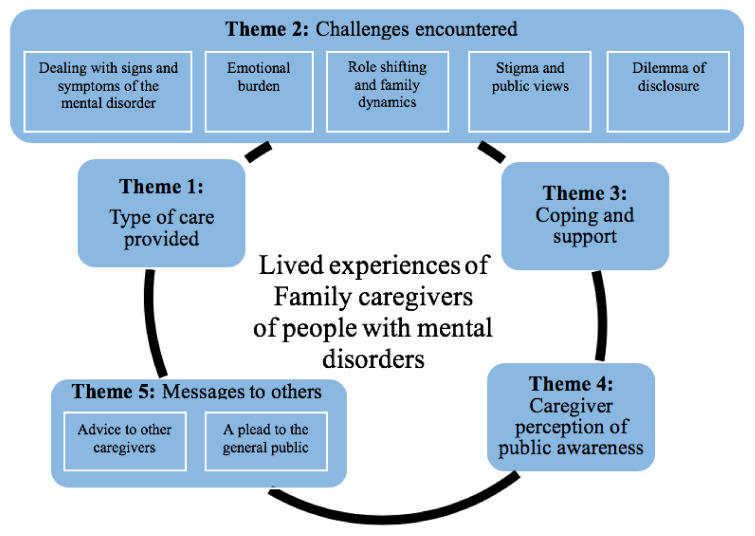
Diagrammatic representation of the main and sub-themes found in this study.

Five main themes and seven subthemes were extracted from the interviews: (1) Type of care provided, (2) Challenges encountered, (3) Coping and support, (4) Caregiver perception of public awareness of mental disorders, and (5) Messages to others. Figure 1 provides a visual depiction of the themes and their interrelated nature (Figure 1). 

### 3.2. Theme 1: Type of Care Provided

The type of care provided by caregivers differed according to the severity of the mental disorder and age of the individual. Severe mental disorders, such as Alzheimer’s, intellectual disabilities, and schizophrenia, were found to require complete holistic care, as explained by a caregiver of an individual with an intellectual disability: “We are taking care of her basic needs, from going to the toilet to feeding her … everything from A to Z” (C12). Disorders such as depression and bipolar were considered less burdensome, in the sense that people with these types of disorders only required partial physical assistance and more spiritual, psychological, and emotional support. A as one of the caregivers reported: “I was the psychological and spiritual supporter… I used to say to him things like ‘you are strong and you are capable of overcoming this crisis’” (C4). A different caregiver shared: “I do not provide her with that much care, but I do try to make her laugh, and feel better and get her out of her mood” (C3). Some participants also reported offering care in unique forms, such as being the mediator of the family: “I was the dove of peace among all in order to make him comfortable, because he would get unwell when his wife gets mad at him. He used to call me to come and calm everyone down and help resolve the problems because they were always in a state of stress” (C4). Care often extended beyond the immediate individual with the mental illness to practical support for other family members, for example providing childcare when the individual was going through an episode or treatment. “I would look after her children because it was hard for her to take care of them while she was unwell, I will always be there for her… supporting her psychologically... she feels better when she knows that her children are taken care of” (C1). A minority of the participants felt they had to employ a paternalistic approach within their caregivers: “I even sometimes would have to treat him like a child. I would tell him ‘you are a hero; you can do it.’ I was encouraging him so that he did not quit his job” (C2). Another caregiver reported: “She is like a baby, like a child, she has to be taught how to eat without making sounds and how to walk, my mother teaches her the basics like a child” (C6).

### 3.3. Theme 2: Challenges Encountered

Caring for people with mental disorders put caregivers under various stressors, as they have faced different challenges that have affected both themselves and the care that they provide. Five sub-themes based on these different challenges were retrieved from the interviews: (i) Dealing with signs and symptoms, (ii) emotional burden, (iii) role shifting and family dynamics, (iv) stigma and public views, and (v) disclosure dilemma. 

#### 3.3.1. Subtheme i: Dealing with Signs and Symptoms of the Disorder

Dealing with the signs and symptoms of mental disorders often caused heartache among caregivers, as explained by a participant: “You can’t imagine how hard it is to see your father or your mother, the people who love you the most, no longer remember you! So, there is no reason for living” (C10). Caregivers also mentioned how inadequate education and training on how to care and manage the signs and symptoms posed a significant challenge. Caregivers commonly reported not knowing if the care they were providing was the right type of care, generating internal self-conflict: “Sometimes we do not know how to deal with him… I do not know… I feel it is wrong to deal with him as a normal person, but at the same time, we have to deal with him as a normal person, I really do not know how to deal with him, it is very hard” (C7). At times, dealing with the signs and symptoms of the mental disorders put the caregivers at risk of physical injury, aggression, or violence; as one of the participants noted: “The challenge that I was suffering from was her violence. She hit me!” (C6). Even without physical violence, caregivers have faced physical burdens due to the type of care itself. When participants were asked about whether the care they provided affected them or their lives in any way, the majority acknowledged a substantial impact on their lives: “It’s exhausting, maybe you can do it by yourself, but it is better to seek help; if it was my decision, I would hire a nurse for him!.” (C11). However, the impact of the challenges could also have a motivating effect, such as in the case of one caregiver who reported that the experience had impacted her choice of career: “It affected my choice of specialty in medical school because of something that happened to me in my personal life” (C8). Most of the caregivers reported issues regarding medication compliance and non-adherence among the relatives they were caring for. One of the caregivers said: “Sometimes when he feels comfortable and stable, he stops the medication. He always says: I don’t need it anymore” (C4). Another caregiver faced the same situation: “When she feels sick, she takes her medication, until she starts to feel fine then she stops taking it causing her to relapse” (C1). Some of the caregivers did not face this type of problem, as they reported that their relative was independent and capable of adhering to their therapeutic plan: “She was taking her medications by herself and she doesn’t have any problem in remembering her medications, she even reminds us about our medication time” (C3). A final challenge existed where participants had sole care. While the majority of the participants reported a shared responsibility of care between family members: “Me and my older sister were taking care of her” (C12), the few with complete responsibility described it as an obligatory assignment. This could lead to other types of challenges that were mentioned earlier. “When I was in the first year of college, it affected me a lot because I was with him (his father) in the hospital because he broke his hip and I needed be there with him all the time, and I could not find the time to study” (C10). 

#### 3.3.2. Subtheme ii: Emotional Burden

All of the participants reported that the most severe challenge that they faced is the emotional burden, which has had multiple effects on them and the care that they provide. Participants reported feeling shock, severe sadness, depression, exhaustion, helplessness, and even an inability to comprehend the situation altogether. This was exemplified by the following caregiver: “It was hard when I saw her crying, I felt like someone is breaking me and tearing me into pieces” (C13). Another participant added: “I was in psychological distress myself, I felt like I was stuck in a whirlpool, a great big whirlpool… I cried very hard, it is such a difficult test from God… to have your loved ones stricken by this illness (the caregiver was crying)” (C1). At times, the emotional burden was so severe the caregiver themselves were subjected to developing mental health problems: “In 2008, I was diagnosed with depression” (C10). Most of the caregivers mentioned feeling “helplessness,” as they did not know how to deal with their loved ones: “Mmm...sometimes we don’t know how to deal with him, I feel like we shouldn’t treat him like a sick person or a normal one…” (C7).

#### 3.3.3. Subtheme iii: Role Shifting and Family Dynamics

The homeostasis of the family structure is shaken during episodes of mental instability. The caregiver may be in charge of different tasks and may be responsible for playing additional roles that exacerbate the different types of burdens that they face. Some participants mentioned that they faced challenges related to the shifting of roles within the family, which, in turn, affected their own social and personal lives: “When you are responsible for supporting a family to become balanced and able to overcome a crisis, this will take a lot of effort” (C4). Another participant experienced having to take on the role of being the head of the family while he was still of a young age: “The court obligated me to be my father’s guardian when I was just 19” (C10).

#### 3.3.4. Subtheme iv: Stigma and Public Views

Another reality highlighted by caregivers was stigma, as public perception is generally negative, with unfair or unsubstantiated fears and beliefs about individuals with mental disorders. The majority of the caregivers were in agreement that the general public described people with mental disorders as crazy, as one of the caregivers pointed out: “They [members of the public] do not have any idea, and for them the mentally ill is ‘crazy,’ and they do not have a life and they are incapable of doing anything” (C9). The public perception could also extend within the walls of the family. One of the caregivers related her experience of stigma and discrimination from a family relative: “One of our relatives came to visit us once with his wife, they were newly married, he asked us to hide my sister, I didn’t like what he said, this is her home. Why should we hide her? We kept her in her room, watching TV and got her dressed and prepared just in case anyone saw her” (C12). Another caregiver also voiced her concern with regard to exploitation of the individual’s vulnerability: “She was not helping him, she was taking advantage of his illness and using him to do what she wants when he is sick she would ask him to buy for her lots of things because she knew that he doesn’t have control” (C4).

#### 3.3.5. Subtheme v: Dilemma of Disclosure

With regard to disclosure, caregivers were in self-conflict as to whether disclosing the diagnosis of their relative to others was acceptable or not. Reasons for self-conflict included not wanting to be pitied and a sense of preserving privacy. For example, one of the caregivers believed that it was a personal matter, stating: “I did not have to tell them (distant relatives); I did not tell them because this is my problem and no one else” (C1). Another explained that she would not tell anyone: “I wouldn’t go around and advertise and tell people; I wouldn’t complain to anyone” (C4). The caregivers seemed to agree about disclosing to close blood relatives, such as siblings, parents, and spouses. However, fear of being stigmatized meant they were less willing to disclose to distant relatives and other acquaintances. “I would feel better if no one knew about her mental disorder… it is okay if the close ones (close relatives) know ... but the rest, no, it is better that no one knows” (C6).

### 3.4. Theme 3: Coping and Support

The notion of coping was varied among caregivers. The majority of the study participants mentioned acceptance and religious practices as effective coping mechanisms. One of the caregivers commented: “All praise to God, we accept it because we know God is generous, eventually, we can’t escape from fate, God will be above everything” (C1). They also believed in the importance of the caregivers being close to God: “I would seek support from God only, because no one will stand beside you except your Lord. He is the one who afflicted me and so he is the one who is able to heal, God has tested me and I accept it. God is great and he is compassionate … I have a strong faith in God that he is able to heal her” (C1). Some of the participants described their relatives who were affected by the mental disorder as a mercy from God: “To be honest, there was no pressure on us. Our father was telling us: ‘She is the cause of mercy and blessings in our life,’ and when we would feel tired, he would take over and do all the care by himself” (C12). Other caregivers chose to take an educational approach to coping by learning more about the disorder and how to deal with the individual: “I attended training courses about autism and people with special needs, I also communicated with a specialized doctor and behavioral therapists” (C5). One of the participants talked about how learning and gaining new information about their relative’s mental illness helped to increase her awareness: “…mainly I was reading about it, the more I learned, the less fearful I became” (C8). Only a couple of caregivers utilized less effective coping mechanisms, such as avoidance: “Ignoring, ignoring! I did not talk to her for one year and I left the room because we were roommates” (C6). The ability to cope was often linked to access to additional support, either psychological support, support groups for the individual, or practical resources and services. One of the caregivers expressed her need for support by saying: “I needed to know more information about the illness, I was reading from the internet but I needed a trusted source, and I feel like it is helpful for him to engage in support groups but we do not have any here” (C7).

### 3.5. Theme 4: Caregiver Perception of Public Awareness of Mental Disorders

Participants were asked to share their perceptions of the public awareness about mental disorders in Saudi Arabia. Although the caregivers had varying opinions, the majority agreed on the misconceptions of society regarding the causes, treatment, and care of mental disorders. They also mentioned stigma and the underestimation of people with mental disorders and their capabilities. The most common related misconception was about the causes of mental disorders, as the majority agreed that the Saudi society still associates the causes of mental disorders with magic, the evil eye, and possession: “I feel like all the people think that the evil eye is the cause” (C6). One of the caregivers themselves believed that the main cause of her mother’s sickness was possession: “My mom had fallen unwell, she was possessed by an evil spirit, and she was treated by a sheikh (a religious faith healer), and then she was diagnosed with depression” (C13). The caregivers believed that the first choice of treatment among many would involve seeking help from a sheikh, who would usually treat a person with religious practices involving the reading of Quranic verses: “To be honest with you, we also took him to a sheikh to read [qur’anic verses] over him” (C2). Despite the negative experiences, some caregivers believed that the Saudi public were more understanding and accepting of certain mental disorders over others. For example: “If we were talking about mental disorders such as Alzheimer’s or dementia…the public deal with these disorders as expected and normal disorders, but when you are talking about depression, bipolar, or schizophrenia, they don’t believe that you are sick, they think you are lying or faking it…They say ‘get closer to God, go pray, there is something wrong with your faith,’ they just can’t understand that the problem is organic in nature” (C11). However, in general, the caregivers believed that the public did not have enough knowledge about mental disorders: “I wouldn’t say they are unaware, but they have superficial information about it (mental disorders)” (C8). Additional misconceptions noted by the participants were about treatments and care. Several caregivers noted that the general public believed in the religious treatment of mental disorders by faith healers, or sheikhs, and indeed several of the participants had sought this case as stated previously. One caregiver said: “Some of them think that you must go to a sheikh or so on rather than seek psychiatric treatment” (C11). The specific rationale for this was understood to be a lack of trust in psychiatry and a stigma of shame in seeking psychological help: “…they think it is a shame that we go to a psychiatrist, simply put, they would say: I am not crazy” (C4). One of the caregivers was accepting of their opinion: “I was one of the people who did not believe in psychiatrists” (C3). Most caregivers shared the belief that the public are unaware that people with mental disorders are capable of living fulfilling lives and can be independent and successful people, as society only focuses on their limitations rather than on their strengths. A caregiver explained how her daughter was a successful individual, regardless of her mental disorder: “She is an amazing mother, a wonderful wife, social with her colleagues and her students love her. She has two master degrees and is now doing her PhD” (C1).

### 3.6. Theme 5: Messages to Others

As they discussed their experiences, particularly reflecting on those in the previous theme, a number of key messages were shared that fell within to two subthemes: (i) Advice to other caregivers, and (ii) A plea to the general public.

#### 3.6.1. Subtheme i: Advice to Other Caregivers

Most caregivers shared similar advice regarding care provided to their relatives with mental disorders: “You have to be careful about your words, avoid using words that may hurt them, even if it is a joke” (C7). They also stressed the importance of psychological support, tolerance, and patience: “They shouldn’t treat him as a sick person … and they have to be patient” (C10). One of the caregivers caring for a person with Alzheimer’s shared this message: “Just stay close to them, listen to their stories, even if he told you the same story a thousand time before, honestly their smiles can fix everything. When I saw my father’s smile, I felt safe, happy and in peace, if you lose them you will lose everything” (C10). A few also noted that self-care was key to maintaining effective care provision: “If you want to take care of your mentally ill relative, you have to maintain your psychological status first, you have to be patient, and you have to learn how to deal with the individual” (C6). Another caregiver said: “Make some time for yourself” (C11).

#### 3.6.2. Subtheme ii: A Plea to the General Public

The messages of the caregivers to the public can be summarized as follows: educate yourself, increase mental health services, accept people with mental disorders, and there is no shame in seeking mental health services. Given the extent and range of mental health disorders, caregivers expressed concern that people make an effort to educate themselves on the subject, suggesting different ways people could do this: “Attend online courses, I wish they would take the time to educate themselves on mental health in general, because if you read about it, you will like it and you will feel it’s normal, just like other disorders” (C8). They also offered a compassionate call, encouraging those who need to seek it out and to reduce the surrounding stigma: “I wish that if anyone has the feeling that he or she needs some help, go and look for it, go to a counselor or psychiatrist ... there is no shame in going and seeking help” (C10).

## 4. Discussion

This study provides insight into the unique experiences of Saudi caregivers of people with mental disorders, revealing that the type of care provided to individuals varies depending on the severity of the mental disorder, which is consistent with the results found in the literature [12,26]. There were a number of assumptions about anticipated findings regarding caregiver burden and coping made by the lead researcher based on her expertise and experience in mental health care within the SA context. Namely, that caregivers would have experienced high levels of stigma towards themselves and the people in their care. The analysis drew on a phased team approach to help bracket these assumptions and ensure that the analysis was representative of the caregiver’s experiences rather than confirming assumptions. As it transpired, these assumptions were born out in the data, however, an unanticipated finding was that the majority of stigma came from family members of the caregiver, rather than the wider society.

Within this study, caregivers reported many challenges: dealing with the signs and symptoms of a mental disorder, emotional burden, violence and physical burden, role shifting and disturbed family dynamics, stigma, and public views. Similar challenges—stigma and emotional burden—were found in studies conducted in Taiwan [27], Ghana [28], and Iran [12], which linked emotional burden rates with poor mental health, stress, and psychological distress among caregivers [12,28,29]. In the current study, caregivers reported psychological distress and the development of mental disorders, such as depression. As stated, the stigma faced by the majority of caregivers in the current study was reported to be typically from relatives, rather than society in a wider sense. This was found to be the reason behind the caregivers’ concealment of their relatives’ illness and their hesitation to disclose their diagnosis. The need to conceal and the hesitancy to disclose can be attributed in part to the affiliate stigma experienced by caregivers [30]. This may in part be related to the public’s low mental health awareness, as well as the patronizing and labeling nature of the Arab culture, which tends to be paternalistic toward those with mental disorders and underestimates their abilities and level of functioning [31]. The types of coping strategies reported in the current study were divided according to their effectiveness. Acceptance-, religiosity-, and spirituality-related coping techniques emerged as the main effective coping strategies. The caregivers believed that all hardships experienced would be reduced by praying and seeking refuge from God. In Islamic belief, God’s will is above all and, thus, religion plays an integral role in symptom formation, management, and acceptance [32]. This finding is in line with that of other research conducted in Tanzania and Malaysia, which also reported acceptance, faith, and religious practices to be the most common and effective coping strategies used by caregivers [33,34]. It is likely that the similarity in results between the current study and those conducted in Malaysia and Tanzania is, in part, related to the shared belief between the samples, namely, mostly Muslim and with shared a belief in God and fate. On the other hand, the less effective coping strategies noted by the caregivers in the current study were avoidance, ignoring, and engagement with the person’s non-reality-based thinking (i.e., hallucinations and delusions). These are also reflected in caregivers of people with mental disorders in other countries for example in Zimbabwe, Ethiopia, and India [7,35,36]. It should be noted that the majority of caregiving was provided by women, making them more susceptible to caregiver burdens [16]. Moreover, the lower levels of mental health literacy and the limited knowledge among Saudi caregivers of people with mental disorders put them at higher risk of practicing poor coping techniques [37]. Caregivers were divided in their responses and opinions when asked about the general public’s awareness regarding mental disorders, though numerous misconceptions shared by the public were pointed out by the caregivers under this theme. The majority of caregivers shared the perception that Saudi society still believes that there is a strong relationship between the cause and occurrence of mental disorders and black magic, the evil eye, and possession. A similar finding has been noted in a Malaysian study, where caregivers reported that there was a tendency to seek out traditional faith healers to cure mental disorders, as there is a societal sense of believing in evil spirits and possession [34]. The messages from caregivers to the public and other caregivers were to educate oneself, increase the availability of mental health services, practice acceptance, and seek out help from mental health services without shame. This highlights a need for future research and practice.

### 4.1. Implications for Future Research and Practice

The findings of the current study point to a need for solutions to reduce the burdens and challenges of caregivers of people with mental disorders. Moreover, this study highlights significant implications for mental health professionals and, in particular, nurses. There is a need for mental health nurses to focus not only on the care they provide to people with mental disorders, but to extend their care to include the individual’s primary caregivers. As the above-mentioned results of the current study explain, family caregivers face challenges and experience different types of burdens, and are, in turn, susceptible to the development of psychological disorders themselves. This poses a significant implication for nursing practice that matches the literature, which suggests that nurses have a professional responsibility to provide holistic care to caregivers in the form of counseling, family and supportive therapy, promotion of self-care activities, education on effective coping strategies, and health teaching [38,39]. Moreover, the advocacy, supportive services, and education provided by nurses to caregivers can contribute to the prevention of physical exhaustion and burnout among caregivers, and can contribute to an increase in societal mental health awareness. Based on the collected data, there exists a need to raise public awareness about mental disorders and to accept people with mental disorders and their families. This presents a unique area for future research within the Arabic context to reduce stigma which may not translate cross-culturally [40]. Furthermore, there is a dire need to provide supportive services and education to caregivers to increase their knowledge about how to deal with different mental illnesses. Additionally, there is a need to encourage the engagement of caregivers of people with mental illnesses with the community.

### 4.2. Study Limitations

The use of social media platforms to recruit participants meant that a larger potential sample were notified about the study, however, we note that caregivers experiencing burden may not be accessing such platforms, particularly in times of stress. There is also the potential for sampling bias, as those who replied and participated were motivated to do so. As such, the findings presented in this paper provide insight into family caregivers experiences, which have generated new hypotheses for the study team to further investigate, but cannot be considered representative of all family caregivers with relatives experiencing mental illness.

Finally, these data contain experiences from an Arab culture, building on a limited data set speaking to mental illness in Middle Eastern countries. However, the findings may also have relevance for healthcare workers who are caring for the many families who have emigrated beyond these boarders. To this end, the research team chose to translate the data into English to enable greater scope for dissemination. Though it should be noted that some cultural nuances in the language used by participants may have been lost in the translation process. This speaks to a wider limitation within the scientific community, and the need for increased duel-language publications.

## 5. Conclusions

Our findings suggest that caregivers experience distinct types of challenges that can disrupt their biopsychosocial integrity, such as dealing with the symptoms of mental disorders and possible violence, physical burnout, emotional burden, impaired family dynamics and role shifting, as well as stigma related to public views. Additionally, the results emphasize the need to identify the different types of care, the effectiveness of the coping strategies of caregivers, and the types of support they require. Furthermore, this research highlights the advice and messages that caregivers would like to share with other caregivers and members of the public in order to correct their misconceptions about the causes and treatment of mental and psychological disorders. Finally, we mentioned the application of nursing care, which should not only focus on people with mental disorders, but should also be directed toward their caregivers, who face many challenges and experience different types of burdens.

## Figures and Tables

**Table 1:** Study participants’ characteristics.

Caregiver Identifier	Age	Gender	Marital Status	Occupational Status	Educational Level	Relationship to Individual	Family Member’s Mental Disorder	Family History of Mental Disorders	Caregiver Role	Diagnosis Period
C1	65	Female	Married	Housewife	Diploma	Daughter	Bipolar, depression, and obsessive-compulsive disorder	Positive	Not shared	9 years
C2	42	Female	Married	Housewife	Bachelor’s	Husband	Depression	Negative	Shared	1 year
C3	47	Male	Married	Employed	High school	Mother	Depression	Positive	Shared	40 years
C4	65	Female	Married	Housewife	Diploma	Brother	Bipolar	Positive	Not shared	30 years
C5	34	Male	Married	Employed	Bachelor’s	Son	Autism	Negative	Shared	Since birth
C6	22	Female	Single	Student	Bachelor’s	Sister	Psychosis	Positive	Shared	2 years
C7	22	Female	Single	Student	Bachelor’s	Brother	Bipolar type 2	Positive	Shared	5 years
C8	21	Female	Single	Student	Bachelor’s	Sister	Bipolar	Unknown	Shared	10 years
C9	21	Female	Single	Student	Bachelor’s	Mother	Depression	Positive	Shared	7 years
C10	24	Male	Single	Student	Bachelor’s	Father	Alzheimer’s	Positive	Shared	5–6 years
C11	21	Female	Single	Student	Bachelor’s	Uncle	Schizophrenia	Negative	Shared	Since birth
C12	39	Female	Married	Employed	High school	Sister	Intellectual disability	Negative	Shared	Since birth
C13	22	Female	Single	Student	Bachelor’s	Mother	Depression	Positive	Not shared	10 years
